# Relationship between a perioperative intravenous fluid administration strategy and acute kidney injury following off-pump coronary artery bypass surgery: an observational study

**DOI:** 10.1186/s13054-015-1065-8

**Published:** 2015-09-28

**Authors:** Ji-Yeon Kim, Kyoung-Woon Joung, Kyung-Mi Kim, Min-Ju Kim, Joon-Bum Kim, Sung-Ho Jung, Eun-Ho Lee, In-Cheol Choi

**Affiliations:** Department of Anesthesiology and Pain Medicine, Asan Medical Center, University of Ulsan College of Medicine, 388-1, Pungnap 2-dong, Songpa-gu, Seoul 138-736 Korea; Department of Clinical Epidemiology and Biostatistics, Asan Medical Center, University of Ulsan College of Medicine, Seoul, Korea; Department of Thoracic and Cardiovascular Surgery, Asan Medical Center, University of Ulsan College of Medicine, Seoul, Korea

## Abstract

**Introduction:**

Saline-based and hydroxyethyl starch solutions are associated with increased risk of renal dysfunction. In the present study, we tested the hypothesis that balanced solutions and a limited volume of hydroxyethyl starch solution (renal protective fluid management [RPF] strategy) would decrease the incidence of postoperative acute kidney injury (AKI) and improve clinical outcomes in patients undergoing off-pump coronary artery bypass graft surgery (OPCAB).

**Methods:**

We investigated 783 patients who underwent elective OPCAB. All patients who underwent OPCAB between 1 January 2010 and 4 July 2012 formed the control group and were given intravenous fluids with saline-based solutions and unlimited volumes of colloid solutions. All patients who underwent OPCAB between 5 July 2012 and 31 December 2013 formed the RPF group and were given intravenous fluids with RPF. The primary outcome was the incidence of postoperative AKI. Secondary outcomes included the incidence of severe AKI, requirement for renal replacement therapy, renal outcome at the time of discharge, and other clinical outcomes.

**Results:**

Postoperative AKI occurred in 33 patients (14.4 %) in the RPF group compared with 210 patients (37.9 %) in the control group (*P* < 0.001). The incidences of severe AKI and persistent AKI after OPCAB were significantly lower, and the postoperative extubation time and duration of hospital stay were significantly shorter, in patients in the RPF group than in those in the control group. After adjustment by multivariate regression analyses and inverse probability of treatment weighting adjustment, the RPF group was independently associated with a lower incidence of postoperative AKI, severe AKI, and persistent AKI and a shorter postoperative extubation time and duration of hospital stay.

**Conclusions:**

The RPF strategy is associated with a significantly decreased incidence of postoperative, severe, and persistent AKI in patients undergoing OPCAB, although residual confounding may be present.

**Electronic supplementary material:**

The online version of this article (doi:10.1186/s13054-015-1065-8) contains supplementary material, which is available to authorized users.

## Introduction

Acute kidney injury (AKI) is a well-known, common, and serious complication of coronary artery bypass graft surgery. AKI is associated with a prolonged hospital stay and increased morbidity and mortality, even for patients who do not progress to renal failure [1–3]. In clinical studies, the incidence of AKI is approximately 18 % and the incidence of new-onset renal failure requiring renal replacement therapy (RRT) is approximately 2–6 % after cardiac surgery [4, 5]. Because there are no effective treatments for AKI, the current focus of clinicians is on prevention and risk factor management of this condition.

Perioperative fluid management can affect postoperative renal function. Normal saline solution is the most commonly used intravenous solution in perioperative fluid management [6]. However, recent clinical studies have shown that excessively large volumes of saline-based solutions may cause hyperchloremic acidosis, which can lead to decreased renal blood flow and poor clinical outcomes [7–11]. In a previous human study, the intravenous infusion of normal saline reduced renal blood flow velocity and renal cortical tissue perfusion [12]. In contrast, other studies have shown that balanced crystalloid and colloid solutions can prevent the development of hyperchloremic metabolic acidosis and increase the mean level of renal cortical tissue perfusion compared with saline-based solutions [13–15]. The use of balanced solutions has also been associated with a reduced risk of renal dysfunction in surgical and critically ill patients [16, 17]. Thus, balanced salt solutions containing physiologic levels of chloride and buffer may offer clinical benefits over saline-based solutions.

Hydroxyethyl starch (HES) solution is one of the most commonly used synthetic colloids for correcting hypovolemia during surgery. Compared with crystalloid solutions, colloid solutions are generally considered to be more effective volume expanders because of their smaller volume of distribution [18]. However, several recent reports have suggested that the administration of HES solution is associated with an increased risk of renal dysfunction, coagulation abnormality, and mortality [19–21]. These adverse effects of HES seem to increase with higher doses [20, 22]. Thus, given the safety issues of saline-based and HES solutions for renal function, perioperative fluid management using balanced solutions and limited volumes of HES solution (renal protective fluid management [RPF] strategy) may have beneficial effects on renal function and other clinical outcomes.

The purpose of this study was to investigate the effect of an RPF strategy on the postoperative renal function and clinical outcomes in patients who underwent off-pump coronary artery bypass graft surgery (OPCAB). We hypothesized that an RPF strategy would be associated with a decreased incidence of postoperative AKI and improved clinical outcomes.

## Material and methods

### Patient population

This retrospective observational study was conducted with all patients aged 20 years or older who underwent OPCAB at Asan Medical Center, Seoul, Korea, between 1 January 2010 and 31 December 2013. All clinical data of the study patients, including demographics, laboratory data, and perioperative fluids, were acquired from the Asan Medical Center Coronary Artery Bypass Surgery and Anesthesia Database and by a retrospective review of the computerized patient record system (Asan Medical Center Information System Electronic Medical Record) [23]. Exclusion criteria included emergency or urgent surgery (n = 37), preexisting end-stage kidney disease requiring dialysis (n = 32), prior nephrectomy or kidney transplantation (n = 4), and any concomitant cardiac surgery besides OPCAB (n = 2). Two patients in the RPF group receiving saline-based solution for hyponatremia were also excluded. This study was approved by the local ethics committee (Asan Medical Center Institutional Review Board, Seoul, Republic of Korea, protocol number 2014-0697) and performed in accordance with Strengthening the Reporting of Observational Studies in Epidemiology guidelines [24]. The board waived the need for informed consent.

This retrospective study included two cohorts that were defined on the basis of the type and amount of fluid received during surgery and in the immediate postoperative period (i.e., within 48 h after surgery). On 5 July 2012, we changed the perioperative fluid management strategy from a saline-based solution and unlimited amounts of HES solution to a balanced solution and limited amounts of HES solution (RPF strategy). We defined the control group as patients who underwent OPCAB during the control period (between 1 January 2010 and 4 July 2012) and the RPF group as patients who underwent OPCAB during the renal protection period (between 5 July 2012 and 31 December 2013). This control period was chosen because surgical and perioperative care were similar, except for the perioperative fluid management strategy.

For fluid management, all patients in the control group were given intravenous fluids with a saline-based solution and unlimited amounts of colloid solution during surgery and in the immediate postoperative period. The saline-based solution comprised chloride-rich fluids and included 0.9 % saline (chloride concentration 150 mmol/L; JW Pharmaceutical, Seoul, Korea) and 6 % HES 130/0.4 (chloride concentration 154 mmol/L, Voluven®; Fresenius Kabi, Bad Homburg, Germany). In contrast, all patients of the RPF group were given intravenous fluids with a balanced solution during surgery and a limited amount of colloid solution during surgery and in the immediate postoperative period (cumulative amount <30 ml/kg body weight). The balanced solution comprised chloride-restrictive fluids and included Plasma Solution-A (chloride concentration 98 mmol/L; CJ HealthCare, Seoul, Korea) and 6 % HES 130/0.4 (chloride concentration 110 mmol/L, Volulyte®; Fresenius Kabi). In the immediate postoperative period, the patients in the RPF group received balanced colloid and saline-based crystalloid (0.9 % saline) solutions.

### Intraoperative and postoperative management

Our perioperative management strategies have been described in detail previously [23]. Briefly, anesthesia was maintained with a continuous infusion of propofol and remifentanil using a target-controlled infusion pump (Orchestra Base Primea; Fresenius Vial, Brézins, France). To maintain cardiac preload, patients were administered intravenous fluids, and those with a hemoglobin concentration <8 g/dl were administered packed red blood cells (pRBCs). Despite optimization of the circulating blood volume, patients with low blood pressure or cardiac output received agents such as phenylephrine, dopamine, or norepinephrine. A cell salvage device (autoLog Autotransfusion System; Medtronic, Minneapolis, MN, USA) was used in all patients, and salvaged blood was reinfused before the end of surgery. All surgical procedures were conducted by three cardiac surgeons highly experienced in OPCAB. Median sternotomy was performed in all patients, and complete myocardial revascularization with either arterial or saphenous vein grafts was the primary surgical goal for each patient. At the end of surgery, patients were transferred to the intensive care unit (ICU). Patients were extubated when their hemodynamics were stable, after which they breathed spontaneously and achieved adequate blood gases. Fluid management consisted of infusion of 5 % dextrose with additional normal saline or colloid solution to maintain normovolemia. pRBCs were transfused to maintain hemoglobin >9 g/dl, and fresh frozen plasma, platelet concentrate, or cryoprecipitate was transfused to correct blood coagulation. Patients were discharged from the ICU to the general ward when their clinical status was stable and further ICU monitoring and care were not required. Perioperative fluid management is further described in Additional file 1: Supplementary methods.

### Definition of outcomes

The primary outcome of this study was the incidence of postoperative AKI. The occurrence of AKI after surgery was determined according to Kidney Disease: Improving Global Outcomes (KDIGO) criteria (increase in serum creatinine by ≥0.3 mg/dl within 48 h of surgery or increase in serum creatinine to ≥1.5 times baseline within 7 days of surgery) [25]. The diagnosis of AKI was based on the highest serum creatinine concentration measured during the first 7 days after surgery compared with the baseline serum creatinine concentration, defined as the last concentration measured before surgery. Urine output was not used, because the data were recorded inconsistently among the study patients and may have been affected by diuretic use. AKI was also staged for severity according to the following KDIGO criteria:

- Stage 1: increase in serum creatinine by ≥0.3 mg/dl or 1.5 to 1.9 times baseline
- Stage 2: increase in serum creatinine 2.0 to 2.9 times baseline
- Stage 3: increase in serum creatinine 3.0 or more times baseline or an increase in serum creatinine ≥4.0 mg/dl or initiation of RRT

Secondary outcomes were the incidence of severe AKI (KDIGO stage 2 or higher), requirement for RRT, renal outcome at the time of discharge, time to extubation following surgery, lengths of ICU and hospital stay after surgery, and death due to any cause occurring during the initial hospital stay after surgery. RRT was defined as any use of intermittent hemodialysis or continuous venovenous hemodiafiltration. Renal outcome at the time of discharge was determined by comparing the discharge serum creatinine level to the baseline serum creatinine. Complete renal recovery was defined as a discharge serum creatinine level <1.5 times baseline. Persistent AKI was defined as a discharge serum creatinine >1.5 times baseline or a need for RRT at discharge. Serum chloride levels were measured preoperatively and upon arrival at the ICU. The maximal cardiovascular component of the Sequential Organ Failure Assessment (SOFA-C) score observed within the first 24 h after surgery was used to evaluate postoperative cardiovascular function [26].

### Statistical analysis

Descriptive statistics are reported as numbers and percentages for categorical variables and means with standard deviations for continuous variables. To assess associations between patients’ characteristics and clinical factors, univariate analyses were performed using χ^2^ or Fisher’s exact test for categorical variables and Student’s *t* test or the Mann–Whitney rank-sum test for continuous variables. Univariate and multivariable logistic regression analyses were conducted to investigate the effect of an RPF strategy on postoperative AKI. All variables in Table 1 were tested, and variables with *P* < 0.1 in univariate analyses (i.e., age, diabetes mellitus, hypertension, congestive heart failure, cerebrovascular disease, preoperative estimated glomerular filtration rate <60 ml/min/1.73 m^2^, logistic EuroSCORE [European System for Cardiac Operative Risk Evaluation], preoperative hematocrit, serum creatinine, albumin, uric acid, coronary angiography <5 days, use of angiotensin-converting enzyme inhibitor or angiotensin receptor blocker, use of diuretics, anesthesia time) were entered into the multivariable logistic model. Because perioperative blood product transfusion and maximal SOFA-C score could be associated with the postoperative AKI (i.e., potential confounders), we also adjusted these variables in the main analyses. The final models were determined by backward elimination procedures with *P* < 0.05 as model retention criteria.

**Table 1:** Baseline and intraoperative characteristics

Variable	Control group	RPF group	*P* value
Number of patients	554	229	
Demographics			
Age (yr)	63.9 ± 9.2	63.7 ± 9.4	0.852
Male sex	431 (77.8)	179 (78.2)	0.910
Clinical characteristics			
Body mass index (kg/m^2^)	24.6 ± 3.1	24.6 ± 3.1	0.706
EuroSCORE (logistic)	3.6 ± 3.6	3.5 ± 5.1	0.050
Medical history			
Diabetes mellitus	263 (47.5)	99 (43.2)	0.279
Hypertension	365 (65.9)	151 (65.9)	0.988
Smoker, current	174 (31.4)	58 (25.3)	0.090
Previous myocardial infarction	69 (12.5)	43 (18.8)	0.022
Congestive heart failure	16 (2.9)	12 (5.2)	0.107
s/p PTAC c stent	108 (19.5)	50 (21.8)	0.458
Cerebrovascular disease	145 (26.2)	44 (19.2)	0.038
Peripheral vascular disease	61 (11.0)	18 (7.9)	0.183
eGFR <60 ml/min/1.73 m^2^	81 (14.6)	35 (15.3)	0.812
Liver disease	25 (4.5)	2 (0.9)	0.011
Left main coronary artery disease	128 (23.1)	48 (21.0)	0.513
Coronary angiography <5 days	177 (31.9)	78 (34.1)	0.566
Laboratory data			
Hematocrit (%)	38.5 ± 4.8	38.7 ± 5.4	0.710
Creatinine (mg/dl)	1.0 ± 0.4	1.0 ± 0.4	0.469
Total bilirubin (mg/dl)	0.8 ± 0.3	0.6 ± 0.3	<0.001
Albumin (g/dl)	3.9 ± 0.4	3.8 ± 0.5	0.105
Uric acid (mg/dl)	5.5 ± 1.5	5.6 ± 1.6	0.745
Left ventricular ejection fraction (%)	56.4 ± 10.0	57.1 ± 10.5	0.165
Medication			
ACEI or ARB	249 (44.9)	105 (45.9)	0.817
β-blocker	381 (68.8)	152 (66.4)	0.513
Calcium channel blocker	416 (75.1)	159 (69.4)	0.103
Insulin	127 (22.9)	43 (18.8)	0.200
Oral hypoglycemic agent	209 (37.7)	85 (37.1)	0.873
Statin	431 (77.8)	202 (88.2)	0.001
Aspirin	418 (75.5)	201 (87.8)	<0.001
Clopidogrel	298 (53.8)	146 (63.8)	0.010
Diuretics	104 (18.8)	43 (18.8)	0.999
Intraoperative data			
Anesthesia time (min)	295 [267–335]	300 [275–340]	0.151
Operation time (min)	228 [200–267]	230 [200–262]	0.882
Grafts per patient (n)	3 [2–4]	3 [2–3]	0.015

*RPF* renal protective fluid management, *EuroSCORE* European System for Cardiac Operative Risk Evaluation, *PTCA c stent* percutaneous transluminal catheter angioplasty with stent insertion, *eGFR* estimated glomerular filtration rate, *ACEI* angiotensin-converting enzyme inhibitor, *ARB* angiotensin receptor blocker

Data are expressed as number of patients (%), mean ± standard deviation, or median [interquartile range]

To reduce the impact of treatment selection bias and potential confounding in an observational study, we also performed rigorous adjustment for significant differences in patient characteristics by use of a weighted logistic regression model or a weighted linear regression model using inverse probability of treatment weighting (IPTW). With that technique, weights for patients in the RPF group were the inverse of (1 − propensity score), and weights for patients in the control group were the inverse of the propensity score. The propensity scores were estimated by multiple logistic regression analysis. To create the propensity score, all prespecified covariates were included in the full non-parsimonious models for the RPF group versus the control group. A list of all variables is provided in Additional file 1: eTable S1.

In addition, univariate and multivariate analyses (stepwise selection method for the linear regression model or backward elimination method for the logistic regression model) were performed to determine which of the independent variables were related to the secondary outcome variables. In sensitivity analyses, we explored the effects of additional adjustment for potential confounding variables on the estimated relationship between RPF strategy and AKI. We also examined the effect of an RPF strategy on postoperative AKI in the subgroup with perioperative cumulative amounts of HES <30 ml/kg. Furthermore, we examined the effect of perioperative cumulative amounts of HES as a continuous variable on postoperative AKI. Multivariate logistic or linear regression analyses were used to assess the odds ratios (ORs) or β coefficients with 95 % confidence intervals (CIs) of the relationships between the RPF strategy and outcome variables. The discrimination and calibration abilities of each propensity score model were assessed by using the C statistic and the Hosmer–Lemeshow statistic, respectively.

All reported *P* values are two-sided, and values of *P* < 0.05 were considered to indicate statistical significance. SAS software version 9.3 (SAS Institute, Cary, NC, USA) was used for the statistical analysis.

## Results

**Fig. 1:**
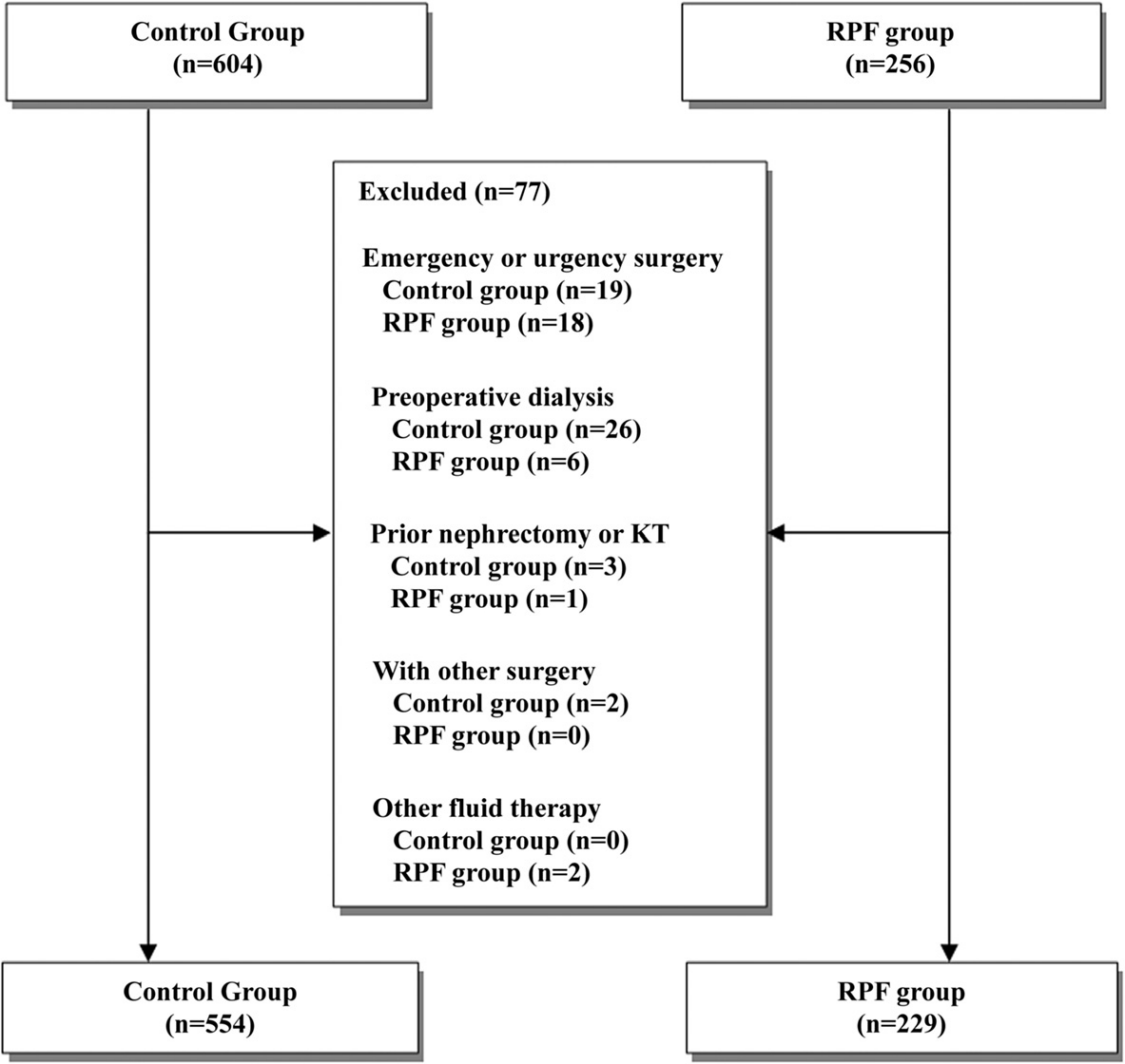
Study flowchart. *RPF* renal protective fluid management, *KT* kidney transplantation

A total of 860 patients underwent OPCAB between 1 January 2010 and 31 December 2013. After the exclusion of patients who met any of the exclusion criteria, 783 patients remained. Of these, there were 554 patients in the control group and 229 in the RPF group (Fig. 1). The baseline and operative characteristics of the study groups are shown in Table 1. There were minor differences between the two groups. Compared with patients in the RPF group, patients in the control group were more likely to have histories of cerebrovascular disease and liver disease. Patients in the control group were also less likely to be receiving statins. Patients in the RPF group received more crystalloid solution (34.3 [25.3–44.5] ml/kg vs. 24.2 [18.4–31.5] ml/kg; *P* < 0.001) and less colloid solution (14.4 [10.1–17.4] ml/kg vs. 18.5 [14.8–22.4] ml/kg; *P* < 0.001) during surgery than the control group (Table 2). The cumulative amount of colloid received during surgery and in the immediate postoperative period was lower in the RPF group than in the control group (16.0 [11.0–20.1] ml/kg vs. 35.0 [27.1–44.7] ml/kg; *P* < 0.001). During surgery and in the immediate postoperative period, patients in the RPF group received fewer units of pRBCs (2.1 ± 1.9 vs. 2.8 ± 2.6; *P* < 0.001), and fewer patients in the RPF group received fresh frozen plasma (33.6 % vs. 47.8 %; *P* < 0.001) and platelet concentrate (14.8 % vs. 32.7 %; *P* < 0.001) than those in the control group. Postoperative percentage body weight gain was higher in the RPF group than in the control group (2.6 ± 2.1 % vs. 1.9 ± 2.2 %; *P* < 0.001).

**Table 2:** Perioperative fluid administration in the study groups

Variable	Control group	RPF group	*P* value
Number of patients	554	229	
Intraoperative data			
Crystalloid (L)	1.6 [1.2–2.0]	2.2 [1.8–2.8]	<0.001
Crystalloid per weight (ml/kg)	24.2 [18.4–31.5]	34.3 [25.3–44.5]	<0.001
Colloid (L)	1.2 [1.0–1.5]	1.0 [0.6–1.0]	<0.001
Colloid per weight (ml/kg)	18.5 [14.8–22.4]	14.4 [10.1–17.4]	<0.001
Colloid-to-crystalloid volume ratio	0.8 [0.6–1.0]	0.4 [0.3–0.6]	<0.001
Cell salvage blood (ml)	237.4 ± 328.0	138.7 ± 208.1	<0.001
Data in intensive care unit			
Colloid (L)	1.0 [0.5–1.5]	0 [0–0.2]	<0.001
Colloid per weight (ml/kg)	16.1 [8.5–24.8]	0 [0–3.1]	<0.001
Cumulative colloid (L)^a^	2.3 [1.8–2.8]	1.0 [0.8–1.5]	<0.001
Cumulative colloid per weight (ml/kg)^a^	35.0 [27.1–44.7]	16.0 [11.0–20.1]	<0.001
Packed red blood cell (unit)^a^	2.8 ± 2.6	2.1 ± 1.9	<0.001
Use of fresh frozen plasma^a^	265 (47.8)	77 (33.6)	<0.001
Use of platelet concentrate^a^	181 (32.7)	34 (14.8)	<0.001
Use of cryoprecipitate^a^	31 (5.6)	6 (2.6)	0.110
Weight gain (%)	1.9 ± 2.2	2.6 ± 2.1	<0.001

*RPF* renal protective fluid management

Data are expressed as number of patients (%), mean ± standard deviation, or median [interquartile range]

^a^Used during surgery and within 48 h postoperatively

**Fig. 2:**
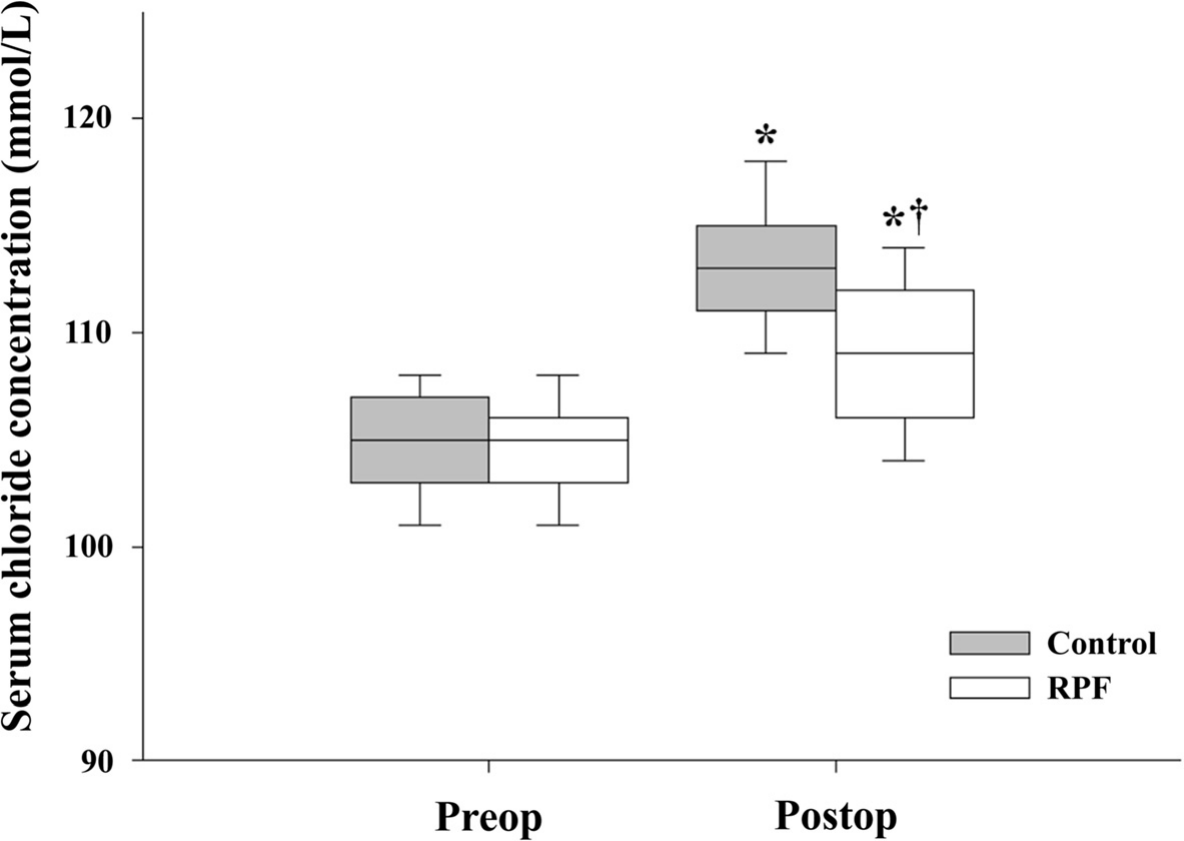
Changes in serum chloride concentration before and after surgery in both groups. Data are expressed as median (interquartile range and range). *P < 0.001 vs. preoperative value. ^†^ *P* < 0.001 vs. control group. *RPF* renal protective fluid management

The preoperative serum chloride level was similar between the two groups (104.4 ± 3.1 mmol/L in the RPF group vs. 104.9 ± 3.2 mmol/L in the control group; *P* = 0.187), but the immediate postoperative serum chloride level was significantly lower in the RPF group than in the control group (108.8 ± 4.3 mmol/L vs. 113.3 ± 3.6 mmol/L, respectively; *P* < 0.001) (Fig. 2).

Postoperative outcomes are shown in Table 3. The postoperative extubation time and the duration of hospital stay were significantly shorter in the RPF group than in the control group, but there was no significant difference between the two groups in the duration of ICU stay or in-hospital mortality. A total of 243 patients (31.0 %) developed AKI after OPCAB surgery. Of these, 192 patients developed AKI within the first 48 h after surgery (Additional file 1: eTable S2). Postoperative AKI occurred in 33 patients (14.4 %) in the RPF group compared with 210 patients (37.9 %) in the control group (*P* < 0.001). The incidence of severe AKI and persistent AKI after OPCAB was also lower in RPF group patients than in the control group (3.5 % vs. 11.4 % [*P* < 0.001] and 1.7 % vs. 10.7 % [*P* < 0.001], respectively). The incidence of RRT was lower in the RPF group, but there was no significant difference between the two groups in this regard.

**Table 3:** Postoperative outcomes in the study groups

	Control group	RPF group	*P* value
Number of patients	554	229	
Acute kidney injury			
RIFLE	212 (38.3)	33 (14.4)	<0.001
AKIN	168 (30.3)	24 (10.5)	<0.001
KDIGO	210 (37.9)	33 (14.4)	<0.001
KDIGO stage ≥2	63 (11.4)	8 (3.5)	<0.001
Renal replacement therapy	12 (2.2)	2 (0.9)	0.345
Persistent acute kidney injury	59 (10.7)	4 (1.7)	<0.001
Extubation time (h)	10.5 [7.5–16.0]	7.5 [5.5–12.3]	<0.001
Intensive care unit stay (h)	47.0 [39.8–50.5]	47.0 [42.5–49.0]	0.695
Maximal SOFA-C score	3.0 [2.0–3.0]	3.0 [0–3.0]	0.181
Hospital stay (days)	8.0 [7.0–10.0]	7.0 [7.0–9.0]	<0.001
In-hospital deaths	7 (1.3)	1 (0.4)	0.512

*RPF* renal protective fluid management, *RIFLE* risk, injury, failure, loss, and end-stage kidney disease classification, *AKIN* Acute Kidney Injury Network classification, *KDIGO* Kidney Disease Improving Global Outcomes classification, *SOFA-C* cardiovascular sequential organ failure assessment in the first 24 h

Data are expressed as number of patients (%) or median [interquartile range]

After adjustment by multivariate regression analyses and IPTW, the RPF group was independently associated with a lower incidence of postoperative AKI (OR 0.31 [95 % CI 0.19–0.50], *P* < 0.001 after multivariate logistic analysis; OR 0.23 [95 % CI 0.15–0.35], *P* < 0.001 after IPTW), severe AKI (OR 0.30 [95 % CI 0.12–0.75], *P* = 0.009 after multivariate logistic analysis; OR 0.12 [95 % CI 0.04–0.36], *P* < 0.001 after IPTW), and persistent AKI (OR 0.20 [95 % CI 0.06–0.67], *P* = 0.008 after multivariate logistic analysis; OR 0.20 [95 % CI 0.08–0.52], *P* = 0.001 after IPTW) (Table 4) (Additional file 1: eTables S3, S4, and S5). The RPF group was also significantly associated with a shorter postoperative extubation time (β −0.28 [95 % CI −0.37 to −0.19], *P* < 0.001 after multivariate analysis; β −0.33 [95 % CI −0.42 to −0.23], *P* < 0.001 after IPTW) and a shorter duration of hospital stay (β −0.11 [95 % CI −0.17 to −0.05], *P* < 0.001 after multivariate analysis; β −0.18 [95 % CI −0.24 to −0.11], *P* < 0.001 after IPTW) (Additional file 1: eTables S6 and S7).

**Table 4:** Impact of renal protection fluid therapy on outcome

	Unadjusted		Multivariable adjusted		IPTW adjusted	
	β (95 % CI)	*P* value	β (95 % CI)	*P* value	β (95 % CI)	*P* value
Extubation time (h)	−0.28 (−0.37, −0.18)	<0.001	−0.28 (−0.37, −0.19)	<0.001	−0.33 (−0.42, −0.23)	<0.001
Intensive care unit stay (h)	0.02 (−0.06, 0.09)	0.682	0.03 (−0.04, 0.10)	0.400	−0.06 (−0.13, 0.02)	0.159
Hospital stay (d)	−0.11 (−0.17, −0.04)	0.001	−0.11 (−0.17, −0.05)	<0.001	−0.18 (−0.24, −0.11)	<0.001
	OR (95 % CI)	*P* value	OR (95 % CI)	*P* value	OR (95 % CI)	*P* value
Acute kidney injury by KDIGO	0.28 (0.18–0.41)	<0.001	0.31 (0.19–0.50)	<0.001	0.23 (0.15–0.35)	<0.001
KDIGO stage ≥2	0.28 (0.13–0.60)	0.001	0.30 (0.12–0.75)	0.009	0.12 (0.04–0.36)	<0.001
Renal replacement therapy	0.40 (0.09–1.79)	0.230	–	–	–	–
Persistent acute kidney injury	0.16 (0.06–0.45)	<0.001	0.20 (0.06–0.67)	0.008	0.20 (0.08–0.52)	0.001
In-hospital death	0.34 (0.04–2.80)	0.318	–	–	–	–

*IPTW* inverse probability of treatment weighting, *CI* confidence interval, *OR* odds ratio, *KDIGO* Kidney Disease: Improving Global Outcomes classification

A significant relationship between RPF strategies and postoperative AKI was consistent with the results of various sensitivity analyses (Additional file 1: eTable S8). There was a significant association between the cumulative volume of HES given and the risk of developing postoperative AKI (Additional file 1: eTable S9).

## Discussion

The major finding of our present study was that the RPF strategy was associated with a significantly lower risk of postoperative AKI in patients who underwent OPCAB. In addition, RPF strategy was found to be significantly associated with a decreased incidence of severe AKI and persistent AKI and a shorter extubation time and hospital stay.

Our results indicate that a perioperative fluid management strategy could affect renal function after OPCAB. This finding is in accordance with previous studies in which researchers reported an association of saline-based solutions and unlimited amounts of HES solutions with renal dysfunction. In recent studies, the use of balanced crystalloid for replacement of fluid losses on the day of major surgery was found to be associated with less postoperative mortality and renal failure requiring dialysis than normal saline [16], and implementation of a chloride-restrictive strategy in a tertiary ICU was found to be associated with a significantly decreased incidence of AKI and use of RRT [17]. In addition, the use of an unlimited amount of HES solution may have been responsible for our present findings. Previous clinical studies have suggested that HES is associated with increased AKI and that higher cumulative doses of HES are associated with greater risk of AKI development [20, 21]. Previous retrospective studies have reported a statistically significant dose-dependent association between intraoperative HES administration and postoperative kidney injury in patients undergoing non-cardiac surgery [19]. Additionally, moderate cumulative doses of HES (>33 ml/kg) may be associated with a higher risk of AKI in surgical ICU patients [22]. Our results also show that the total median cumulative dose of HES in the control group was 35.0 ml/kg and that patients in the control group had a higher incidence of AKI. These findings strongly support the notion that RPF strategy is associated with a decreased incidence of postoperative AKI.

In our present clinical study, we were unable to determine the mechanism by which the saline-based and HES solutions caused an increase in AKI. A possible explanation for this is provided by the renal effect of hyperchloremia. Previous studies have suggested that saline-based solutions contain a supraphysiological concentration of chloride and that hyperchloremia caused by large-volume administration of saline-based solutions may adversely affect kidney function [7, 8]. Hyperchloremic acidosis is associated with renal vasoconstriction, and, in animal models, intrarenal infusion of chloride-rich solutions decreases renal blood flow and the glomerular filtration rate [9, 10, 27]. Two earlier studies with healthy human volunteers found that intravenous infusion of normal saline reduces renal blood flow velocity and renal cortical tissue perfusion, which is not seen with use of a low-chloride solution (Plasma-Lyte 148; Baxter Healthcare, Old Toongabbie, Australia) [12], and that balanced starch increases renal cortical tissue perfusion, an effect not seen with starch in 0.9 % saline [15]. In addition, in elderly surgical patients, the use of balanced crystalloid and colloid solutions prevented the development of hyperchloremic metabolic acidosis compared with normal saline solutions [14]. Our results show that the preoperative serum chloride level was similar between the two study groups but that the immediate postoperative serum chloride level was significantly lower in the RPF group.

Another potential explanation for our present findings could be nephrotoxicity induced by the HES solution. HES solutions are derivatives of maize or potato starch and can be classified according to their molecular weight, concentration, and degree of substitution [22]. Because there is no direct chemical toxicity of HES, the most likely mechanism for HES-induced renal dysfunction would be swelling and vacuolization of tubular cells (“osmotic nephrosis-like lesions”) and tubular obstruction due to the production of hyperviscous urine [28, 29]. Concerns about nephrotoxicity have assumed prominence since the introduction of HES solutions, and several clinical studies have suggested that HES solutions are associated with greater incidence of renal dysfunction and RRT [20, 30]. In line with previous studies, we found that higher cumulative doses of HES were associated with a greater risk of AKI development.

Another pertinent result of our present study is that the RPF group needed less transfusion in the ICU than the control group. This finding is in accordance with previous reports that saline-based solutions may be associated with coagulation abnormalities and that balanced solutions are associated with less coagulation derangement than saline-based solutions [16, 31, 32]. Although no mechanisms have been reported for these putative differences, it is suggested that metabolic hyperchloremic acidosis after administration of saline-based solutions or the small amount of calcium in the buffered electrolyte solution might be important factors [32]. In addition, HES infusions are known to significantly reduce factor VIII, von Willebrand factor, and factor VIII–related ristocetin cofactor due to dilution as well as coating of the platelet. Consequently, there is reduced binding to the platelet membrane receptor glycoprotein Ib (GPIb) and GPIIb/GPIIIa and decreased platelet adhesion [33]. Investigators in several clinical trials have reported that HES is associated with coagulation alterations and increased blood transfusion requirements [20, 21, 34]. Hence, the use of a limited amount of HES would also have affected our results.

The patients in the RPF group in our present investigation had a significantly shorter extubation time and hospital stay. Hyperchloremia in patients undergoing non-cardiac surgery has been associated with a longer hospital stay [11] and hyperchloremia in patients after open abdominal surgery with more time requiring mechanical ventilation [16]. Although we are unable to explain the reasons for such associations on the basis of our present data, previous experimental findings have indicated that hyperchloremia can affect the immune system, alter blood oxygen binding, and induce organ damage [35–38]. Previous studies have also suggested that chloride-restrictive fluid administration and a limited use of HES were associated with a decreased requirement for RRT and mortality [11, 16, 17, 22, 39]. However, we did not find any significant differences in the duration of ICU stay, incidence of RRT, or in-hospital mortality between the two groups. This may be due to a relatively small sample size with low statistical power for identifying their associations. In addition, the RPF group had more positive fluid balance (represented by postoperative weight gain in the present study) than the control group. Given that positive fluid balance has been shown to be linked to poor outcomes in the postoperative period of surgery [40], it is possible that the adverse effects of positive fluid balance could counteract the beneficial effect of RPF strategy on postoperative ICU stay, incidence of RRT, and mortality. Thus, further large studies are required to confirm the clinical implications of our findings.

Researchers in previous studies have speculated that positive fluid balance may affect the diagnosis of AKI because of the dilutional effect of fluid on serum creatinine [41, 42]. Thus, adjustment of creatinine for fluid balance has been proposed for a more accurate assessment of AKI. Unfortunately, we could not calculate corrected serum creatinine for fluid balance, as our database contained no information on fluid output or daily body weight. Therefore, we cannot completely exclude the possibility that differences in fluid balance influenced our results and that the beneficial effect of the RPF strategy on postoperative renal function could be due to unrecognized AKI as a result of the dilution of serum creatinine by the more positive fluid balance. Further studies are warranted to assess this issue.

To our knowledge, this is the first study of the association between RPF strategy and postoperative AKI in cardiac surgery patients. Previous clinical studies have reported that balanced solutions are associated with reduced incidence of postoperative AKI, but few studies have assessed the impact of balanced solutions, including both crystalloids and colloids, on postoperative AKI. In one previous study [17], patients received intravenous fluids with crystalloids and colloids, but these colloids included 4 % succinylated gelatin solution, 4 % albumin in sodium chloride, and 20 % albumin solution and did not include HES. In another study [14], the use of balanced crystalloid and colloid solutions in elderly patients was found to reduce hyperchloremic metabolic acidosis compared with saline-based solutions, but that report did not include an assessment of the effect of balanced solutions on AKI. In our present analyses, patients received intravenous fluids with crystalloid and HES. Hence, we present the first study assessing the impact of balanced solutions with crystalloid and HES on postoperative AKI in patients who underwent OPCAB. Our findings might therefore suggest a perioperative fluid management strategy that could improve the outcome of patients who undergo cardiac surgery.

Our study has several limitations. First, the two study groups were from different time periods, raising the possibility of related confounding factors. For example, growing concerns about AKI might have encouraged anesthesiologists to implement interventions and anesthetics for the RPF group, resulting in better outcomes. Furthermore, although we performed multivariable regression and propensity analyses in an attempt to control bias, the limitations of a non-randomized observational study remain. Because unmeasured variables cannot be controlled by the statistical models presented, it is still possible that residual confounding influenced the results. In other words, it is possible that any differences in renal outcomes between the two groups could be due to unmeasured severity-of-illness differences. Therefore, despite the strong associations between the perioperative RPF strategy and post-OPCAB AKI, given the retrospective observational nature of our analysis, causality could not be determined, and our findings should be considered as hypothesis-generating. Further prospective randomized studies are required to advance understanding of this relationship. Finally, the RPF strategy we used included two different factors: use of balanced solutions and use of a limited amount of colloid solution. We were unable to determine which had more of an impact on our results. However, our findings indicated that these two factors of the RPF strategy were significantly associated with a decreased incidence of postoperative AKI. Accordingly, our present results should be interpreted with caution, and further randomized controlled studies are needed.

## Conclusions

There is a statistically significant and clinically important association between RPF strategy and postoperative AKI in patients undergoing OPCAB. The RPF strategy is associated with a significantly decreased incidence of severe AKI and persistent AKI and a shorter extubation time and hospital stay. The RPF strategy thus appears to improve outcomes in patients undergoing OPCAB and, when used in a perioperative fluid management strategy, can be recommended in patients who show evidence of early AKI or are at risk of AKI.

## Key messages

- Perioperative fluid management strategy could affect postoperative renal function and clinical outcomes.
- The use of balanced solutions and a limited volume of HES solution may have beneficial effects on renal function and other clinical outcomes in patients undergoing off-pump coronary artery bypass graft surgery.

## Additional file

Additional file 1:
**Supplementary method and tables.** Supplementary methods: patient management during study periods. **eTable 1.** Data adjusted with the use of inverse probability weighting. **eTable 2.** Time of diagnosis of acute kidney injury after surgery. **eTable 3.** Multivariable predictors for acute kidney injury after surgery. **eTable 4.** Multivariable predictors for severe acute kidney injury (KDIGO stage 2 or above) after surgery. **eTable 5.** Multivariable predictors for persistent acute kidney injury after surgery. **eTable 6.** Multivariable predictors for delayed extubation after surgery. **eTable 7.** Multivariable predictors for prolonged length of stay in the hospital after surgery. **eTable 8.** Comparison of results for primary analysis of acute kidney injury outcome compared with sensitivity analyses. **eTable 9.** Unadjusted and adjusted relationships between perioperative cumulative amounts (ml/kg weight) of HES and postoperative acute kidney injury. (PDF 252 kb)

## Abbreviations

ACEI: Angiotensin-converting enzyme inhibitor
AKI: Acute kidney injury
ARB: Angiotensin receptor blocker
CI: Confidence interval
eGFR: Estimated glomerular filtration rate
EuroSCORE: European System for Cardiac Operative Risk Evaluation
GP: Glycoprotein
HES: Hydroxyethyl starch
ICU: Intensive care unit
IPTW: Inverse probability of treatment weighting
KDIGO: Kidney Disease: Improving Global Outcomes
OPCAB: Off-pump coronary artery bypass graft
OR: Odds ratio
pRBCs: Packed red blood cells
PTCA c stent: Percutaneous transluminal catheter angioplasty with stent insertion
RIFLE: Risk, injury, failure, loss, end-stage kidney disease
RPF: Renal protective fluid management
RRT: Renal replacement therapy
SOFA-C: Cardiovascular component of the Sequential Organ Failure Assessment

## References

[1] Srisawat N, Lawsin L, Uchino S, Bellomo R, Kellum JA, BEST Kidney Investigators (2010). Cost of acute renal replacement therapy in the intensive care unit: results from The Beginning and Ending Supportive Therapy for the Kidney (BEST Kidney) study. Crit Care.

[2] Bellomo R, Ronco C, Kellum JA, Mehta RL, Palevsky P, ADQI workgroup (2004). Acute renal failure - definition, outcome measures, animal models, fluid therapy and information technology needs: the Second International Consensus Conference of the Acute Dialysis Quality Initiative (ADQI) Group. Crit Care.

[3] Bagshaw SM, George C, Bellomo R, ANZICS Database Management Committee (2007). Changes in the incidence and outcome for early acute kidney injury in a cohort of Australian intensive care units. Crit Care.

[4] Thiele RH, Isbell JM, Rosner MH (2015). AKI associated with cardiac surgery. Clin J Am Soc Nephrol..

[5] Kiers HD, van den Boogaard M, Schoenmakers MC, van der Hoeven JG, van Swieten HA, Heemskerk S (2013). Comparison and clinical suitability of eight prediction models for cardiac surgery-related acute kidney injury. Nephrol Dial Transplant..

[6] Awad S, Allison SP, Lobo DN (2008). The history of 0.9 % saline. Clin Nutr.

[7] Reid F, Lobo DN, Williams RN, Rowlands BJ, Allison SP (2003). (Ab)normal saline and physiological Hartmann's solution: a randomized double-blind crossover study. Clin Sci.

[8] Lobo DN, Stanga Z, Aloysius MM, Wicks C, Nunes QM, Ingram KL (2010). Effect of volume loading with 1 liter intravenous infusions of 0.9 % saline, 4 % succinylated gelatine (Gelofusine) and 6 % hydroxyethyl starch (Voluven) on blood volume and endocrine responses: a randomized, three-way crossover study in healthy volunteers. Crit Care Med.

[9] Wilcox CS (1983). Regulation of renal blood flow by plasma chloride. J Clin Invest..

[10] Hansen PB, Jensen BL, Skott O (1998). Chloride regulates afferent arteriolar contraction in response to depolarization. Hypertension..

[11] McCluskey SA, Karkouti K, Wijeysundera D, Minkovich L, Tait G, Beattie WS (2013). Hyperchloremia after noncardiac surgery is independently associated with increased morbidity and mortality: a propensity-matched cohort study. Anesth Analg..

[12] Chowdhury AH, Cox EF, Francis ST, Lobo DN (2012). Ann Surg.

[13] Base EM, Standl T, Lassnigg A, Skhirtladze K, Jungheinrich C, Gayko D (2011). Efficacy and safety of hydroxyethyl starch 6 % 130/0.4 in a balanced electrolyte solution (Volulyte) during cardiac surgery. J Cardiothorac Vasc Anesth.

[14] Wilkes NJ, Woolf R, Mutch M, Mallett SV, Peachey T, Stephens R (2001). The effects of balanced versus saline-based hetastarch and crystalloid solutions on acid–base and electrolyte status and gastric mucosal perfusion in elderly surgical patients. Anesth Analg..

[15] Chowdhury AH, Cox EF, Francis ST, Lobo DN (2014). A randomized, controlled, double-blind crossover study on the effects of 1-L infusions of 6 % hydroxyethyl starch suspended in 0.9 % saline (Voluven) and a balanced solution (Plasma Volume Redibag) on blood volume, renal blood flow velocity, and renal cortical tissue perfusion in healthy volunteers. Ann Surg.

[16] Shaw AD, Bagshaw SM, Goldstein SL, Scherer LA, Duan M, Schermer CR (2012). Major complications, mortality, and resource utilization after open abdominal surgery: 0.9 % saline compared to Plasma-Lyte. Ann Surg.

[17] Yunos NM, Bellomo R, Hegarty C, Story D, Ho L, Bailey M (2012). Association between a chloride-liberal vs chloride-restrictive intravenous fluid administration strategy and kidney injury in critically ill adults. JAMA..

[18] McIlroy DR, Kharasch ED (2003). Acute intravascular volume expansion with rapidly administered crystalloid or colloid in the setting of moderate hypovolemia. Anesth Analg..

[19] Kashy BK, Podolyak A, Makarova N, Dalton JE, Sessler DI, Kurz A (2014). Effect of hydroxyethyl starch on postoperative kidney function in patients having noncardiac surgery. Anesthesiology..

[20] Brunkhorst FM, Engel C, Bloos F, Meier-Hellmann A, Ragaller M, Weiler N (2008). Intensive insulin therapy and pentastarch resuscitation in severe sepsis. N Engl J Med..

[21] Schortgen F, Lacherade JC, Bruneel F, Cattaneo I, Hemery F, Lemaire F (2001). Effects of hydroxyethylstarch and gelatin on renal function in severe sepsis: a multicentre randomised study. Lancet..

[22] Schabinski F, Oishi J, Tuche F, Luy A, Sakr Y, Bredle D (2009). Effects of a predominantly hydroxyethyl starch (HES)-based and a predominantly non HES-based fluid therapy on renal function in surgical ICU patients. Intensive Care Med..

[23] Lee EH, Yun SC, Chin JH, Choi DK, Son HJ, Kim WC (2012). Prognostic implications of preoperative E/e′ ratio in patients with off-pump coronary artery surgery. Anesthesiology..

[24] von Elm E, Altman DG, Egger M, Pocock SJ, Gøtzsche PC, Vandenbroucke JP, STROBE Initiative (2007). The Strengthening the Reporting of Observational Studies in Epidemiology (STROBE) statement: guidelines for reporting observational studies. Lancet.

[25] Okusa MD, Davenport A (2014). Reading between the (guide)lines—the KDIGO practice guideline on acute kidney injury in the individual patient. Kidney Int..

[26] Vincent JL, de Mendonça A, Cantraine F, Moreno R, Takala J, Suter PM (1998). Use of the SOFA score to assess the incidence of organ dysfunction/failure in intensive care units: results of a multicenter, prospective study. Crit Care Med..

[27] Bullivant EM, Wilcox CS, Welch WJ (1989). Intrarenal vasoconstriction during hyperchloremia: role of thromboxane. Am J Physiol..

[28] Suttner S, Boldt J (2004). [Volume replacement with hydroxyethyl starch: is there an influence on kidney function?]. Anasthesiol Intensivmed Notfallmed Schmerzther.

[29] Hüter L, Simon TP, Weinmann L, Schuerholz T, Reinhart K, Wolf G (2009). Hydroxyethylstarch impairs renal function and induces interstitial proliferation, macrophage infiltration and tubular damage in an isolated renal perfusion model. Crit Care..

[30] Myburgh JA, Finfer S, Bellomo R, Billot L, Cass A, Gattas D (2012). Hydroxyethyl starch or saline for fluid resuscitation in intensive care. N Engl J Med..

[31] Roche AM, James MF, Bennett-Guerrero E, Mythen MG (2006). A head-to-head comparison of the in vitro coagulation effects of saline-based and balanced electrolyte crystalloid and colloid intravenous fluids. Anesth Analg..

[32] Waters JH, Gottlieb A, Schoenwald P, Popovich MJ, Sprung J, Nelson DR (2001). Normal saline versus lactated Ringer's solution for intraoperative fluid management in patients undergoing abdominal aortic aneurysm repair: an outcome study. Anesth Analg..

[33] Datta R, Nair R, Pandey A, Kumar N, Sahoo T (2014). Hydroxyethyl starch: controversies revisited. J Anaesthesiol Clin Pharmacol..

[34] Wilkes MM, Navickis RJ, Sibbald WJ (2001). Albumin versus hydroxyethyl starch in cardiopulmonary bypass surgery: a meta-analysis of postoperative bleeding. Ann Thorac Surg..

[35] Kellum JA, Song M, Almasri E (2006). Hyperchloremic acidosis increases circulating inflammatory molecules in experimental sepsis. Chest..

[36] Kellum JA, Song M, Venkataraman R (2004). Effects of hyperchloremic acidosis on arterial pressure and circulating inflammatory molecules in experimental sepsis. Chest..

[37] Cambier C, Detry B, Beerens D, Florquin S, Ansay M, Frans A (1998). Effects of hyperchloremia on blood oxygen binding in healthy calves. J Appl Physiol..

[38] Pedoto A, Caruso JE, Nandi J, Oler A, Hoffmann SP, Tassiopoulos AK (1999). Acidosis stimulates nitric oxide production and lung damage in rats. Am J Respir Crit Care Med..

[39] Shaw AD, Raghunathan K, Peyerl FW, Munson SH, Paluszkiewicz SM, Schermer CR (2014). Association between intravenous chloride load during resuscitation and in-hospital mortality among patients with SIRS. Intensive Care Med..

[40] Silva JM, de Oliveira AM, Nogueira FA, Vianna PM, Pereira Filho MC, Dias LF (2013). The effect of excess fluid balance on the mortality rate of surgical patients: a multicenter prospective study. Crit Care..

[41] Macedo E, Bouchard J, Soroko SH, Chertow GM, Himmelfarb J, Ikizler TA (2010). Fluid accumulation, recognition and staging of acute kidney injury in critically-ill patients. Crit Care..

[42] Liu KD, Thompson BT, Ancukiewicz M, Steingrub JS, Douglas IS, Matthay MA (2011). Acute kidney injury in patients with acute lung injury: impact of fluid accumulation on classification of acute kidney injury and associated outcomes. Crit Care Med..

