# Supplementary material for: Relationship between a perioperative intravenous fluid administration strategy and acute kidney injury following off-pump coronary artery bypass surgery: an observational study
Source: Crit Care. 2015 Sep 28;19:350. doi: 10.1186/s13054-015-1065-8 (PMC4587764; doi:10.1186/s13054-015-1065-8)
Supplement: Additional file 1: — Supplementary method and tables. Supplementary methods: patient management during study periods. eTable 1. Data adjusted with the use of inverse probability weighting. eTable 2. Time of diagnosis of acute kidney injury after surgery. eTable 3. Multivariable predictors for acute kidney injury after surgery. eTable 4. Multivariable predictors for severe acute kidney injury (KDIGO stage 2 or above) after surgery. eTable 5. Multivariable predictors for persistent acute kidney injury after surgery. eTable 6. Multivariable predictors for delayed extubation after surgery. eTable 7. Multivariable predictors for prolonged length of stay in the hospital after surgery. eTable 8. Comparison of results for primary analysis of acute kidney injury outcome compared with sensitivity analyses. eTable 9. Unadjusted and adjusted relationships between perioperative cumulative amounts (ml/kg weight) of HES and postoperative acute kidney injury. (PDF 252 kb) [file 13054_2015_1065_MOESM1_ESM.pdf]

Relationship between a perioperative intravenous fluid administration strategy and acute kidney injury following off-pump coronary artery bypass surgery: an observational study

Ji-Yeon Kim, M.D., Kyoung-Woon Joung, M.D., Kyung-Mi Kim, M.D., Min-Ju Kim, B.S., Joon-Bum Kim, M.D., Sung-Ho Jung, M.D., Eun-Ho Lee, M.D., In-Cheol Choi, M.D.

Supplementary Methods: Patient management during study periods
eTable 1: Data adjusted with the use of inverse probability weighting.
eTable 2: Time of diagnosis of acute kidney injury after surgery.
eTable 3: Multivariable predictors for acute kidney injury after surgery.
eTable 4: Multivariable predictors for severe acute kidney injury (≥ KDIGO stage2) after surgery.
eTable 5: Multivariable predictors for persistent acute kidney injury after surgery.
eTable 6: Multivariable predictors for delayed extubation after surgery.
eTable 7: Multivariable predictors for prolonged length of stay in hospital after surgery.
eTable 8: Comparisions of results for primary analysis of acute kidney injury outcome compared with sensitivity analyses.
eTable 9: Unadjusted and adjusted relationships between perioperative cumulative amounts (ml/kg weight) of HES and postoperative acute kidney injury.

Supplementary Methods: *Patient management during study periods*

Perioperative therapy for hemodynamic management and monitoring for OPCAB were conducted in the institutional standard manner. All preoperative medications, except for antiplatelet agents, diuretics, angiotensin-converting enzyme inhibitors, and angiotensin II receptor blockers, were continued until the morning of surgery. Before the induction of general anesthesia, an intra-arterial catheter was inserted into the radial artery under local anesthesia. A central venous catheter (MAC™, Arrow International Inc, Reading, PA, USA) and a pulmonary artery catheter (Swan-Ganz CCombo® V CCO/SvO_2/CEDV Thermodilution Catheter, Edwards Lifesciences LLC, Irvine, CA, USA) were inserted through the right internal jugular vein. Using a Vigilance® monitor (Edwards Lifesciences LLC, Irvine, CA, USA), cardiac output and mixed venous oxygen saturation were continuously measured, as were five-lead electrocardiography, pulse oxymetry, capnography, and urine output. Core temperature was kept above 36.0℃ using a forced-air warming system.

During both study periods, the goals of anesthetic management during OPCAB were to maintain hemodynamic stability (mean arterial pressure > 65 mmHg, heart rate > 60 beats/min, central venous pressure of 8-12 mmHg, pulmonary capillary wedge pressure of 12-15 mmHg, urine output > 0.5 ml/kg/h, cardiac index > 2.0 L/min/m^2, and mixed venous oxygen saturation > 70%). Standard hemodynamic management for OPCAB included mainly fluid administration to achieve normovolemia and preset hemodynamic goal in addition to vasopressors or inotropes to prevent excessive fluid administration. Intraoperative fluid management consisted of continuous infusion (4 ml/kg/h) of crystalloid (0.9% saline during control period or Plasma Solution-A during RPF period) as maintenance fluid and additional HES solution (Voluven® during control period or Volulyte® during RPF period) were administered for optimization of circulating blood volume. Postoperative fluid management consisted of continuous infusion of 5% dextrose as maintenance fluid and additional HES solution (Voluven® during control period or Volulyte® during RPF period) were administered to maintain normovolemia.

During control period, unlimited amount of HES solution was used during the perioperative period. However, during RPF period, if doses higher than the maximum cumulative dose (30 ml/kg) of HES solution were required, crystalloid (Plasma Solution-A during intraoperative period and 0.9% saline during postoperative period) or blood product was used as needed.

eTable 1: Data adjusted with the use of inverse probability weighting.

Variable	Control group	RPF group	*P* value
Demographics			
Age(yr)	63.9 ± 9.3	64.2 ± 9.6	0.614
Male gender	429	174	0.310
Clinical characteristics			
Body mass index (kg/m^2)	24.6 ± 3.1	24.7 ± 2.8	0.656
Euroscore (logistic)	3.6 ± 3.8	3.7 ± 4.7	0.862
Medical history			
Diabetes mellitus	250	87	0.232
Hypertension	364	132	0.271
Smoker, current	161	57	0.440
Previous myocardial infarction	80	28	0.639
Congestive heart failure	22	8	0.979
s/p PTAC c stent	115	50	0.484
Cerebral vascular disease	138	55	0.843
Peripheral vascular disease	56	23	0.887
eGFR < 60 mL/min/1.73 m^2	81	29	0.663
Liver disease	19	4	0.201
Left main disease	125	53	0.550
Coronary angiography < 5d	175	73	0.570
Laboratory data			
Hematocrit (%)	38.6 ± 4.8	38.7 ± 5.6	0.772
Creatinine (mg/dl)	1.0 ± 0.4	1.0 ± 0.3	0.785
Total bilirubin (mg/dl)	0.7 ± 0.3	0.8 ± 0.4	0.079
Albumin (g/dl)	3.8 ± 0.4	3.8 ± 0.5	0.443
Uric acid (mg/dl)	5.5 ± 1.5	5.6 ± 1.6	0.813
Left ventricle ejection fraction (%)	56.8 ± 9.8	57.3 ± 9.1	0.450
Medication			
ACEI or ARB	253	82	0.054
Beta blocker	380	146	0.828
Calcium channel blocker	400	164	0.226
Insulin	123	43	0.467

Oral hypoglycemic agent	204	76	0.727
Statin	448	185	0.080
Aspirin	438	178	0.258
Plavix	313	124	0.778
Diuretics	105	43	0.740

Data are expressed as number of patients or mean ± standard deviation.

RPF = renal-protective fluid management; EuroSCORE = European System for Cardiac Operative Risk Evaluation; PTCA c stent = percutaneous transluminal catheter angioplasty with stent insertion; eGFR = estimated glomerular filtration rate; ACEI = angiotensin-converting enzyme inhibitor; ARB = angiotensin receptor blocker.

eTable 2: Time of diagnosis of acute kidney injury after surgery.

	N	< 48 h	48 h-7 days
AKI	243	192 (79.0%)	51 (21.0%)
Control group	210	168 (80.0%)	42 (20.0%)
RPF group	33	24 (72.7%)	9 (27.3%)
Severe AKI	71	27 (38.0%)	44 (62.0%)
Control group	63	24 (38.1%)	39 (61.9%)
RPF group	8	3 (37.5%)	5 (62.5%)
RRT	14	10 (71.4%)	4 (28.6%)
Control group	12	8 (66.7%)	4 (33.3%)
RPF group	2	2 (100%)	0 (0%)

AKI = acute kidney injury; RPF = renal-protective fluid management; RRT = renal replacement therapy.

eTable 3: Multivariable predictors for acute kidney injury after surgery.

Predictor	Odds Ratio	95% CI	*P* value
RPF group	0.310	0.191–0.504	< 0.001
Age (year)	1.023	1.002–1.044	0.032
Diabetes mellitus	1.605	1.124–2.291	0.009
Hypertension	1.551	1.055–2.278	0.025
Coronary angiography < 5d	1.786	1.232–2.588	0.002
Preoperative hematocrit level (%)	0.959	0.920–0.999	0.047
Preoperative serum creatinine level (mg/dl)	2.312	1.413–3.786	0.001
Preoperative serum albumin level (g/dl)	0.486	0.301–0.783	0.003
Maximal SOFAc score ≥ 3	1.463	1.019–2.101	0.039
Packed red blood cell (unit)*	1.207	1.085–1.343	0.001

* used during surgery and within 48 hours postoperatively

CI = confidence interval; RPF = renal-protective fluid management; SOFAc = cardiovascular sequential organ failure assessment in the first 24 hours.

eTable 4: Multivariable predictors for severe acute kidney injury (≥ KDIGO stage2) after surgery.

Predictor	Odds Ratio	95% CI	*P* value
RPF group	0.301	0.121–0.745	0.009
Preoperative hematocrit level (%)	0.901	0.846–0.959	0.001
Preoperative serum albumin level (g/dl)	0.271	0.135–0.543	< 0.001
Preoperative uric acid concentration (mg/dl)	1.256	1.058–1.491	0.009
Maximal SOFAc score ≥ 3	1.364	1.054–1.764	0.018
Packed red blood cell (unit)*	1.278	1.138–1.436	< 0.001

* used during surgery and within 48 hours postoperatively

CI = confidence interval; RPF = renal-protective fluid management; SOFAc = cardiovascular sequential organ failure assessment in the first 24 hours.

eTable 5: Multivariable predictors for persistent acute kidney injury after surgery.

Predictor	Odds Ratio	95% CI	*P* value
RPF group	0.204	0.063–0.665	0.008
Female	1.975	1.037–3.761	0.038
age	1.059	1.021–1.099	0.002
Preoperative serum creatinine level (mg/dl)	3.316	1.856–5.926	< 0.001
Preoperative serum albumin level (g/dl)	0.383	0.188–0.782	0.008
Packed red blood cell (unit)*	1.250	1.137–1.373	< 0.001

* used during surgery and within 48 hours postoperatively

CI = confidence interval; RPF = renal-protective fluid management.

eTable 6: Multivariable predictors for delayed extubation after surgery.

Predictor	Beta	95% CI	*P* value
RPF group	-0.275	-0.366, -0.185	< 0.001
Female	0.148	0.042, 0.254	0.006
Age	0.005	0.001, 0.010	0.048
Body mass index	0.016	0.003, 0.029	0.018
Cerebrovascular disease	0.112	0.015, 0.208	0.023
EuroSCORE (logistic)	0.018	0.006, 0.029	0.003
Coronary angiography < 5d	0.125	0.039, 0.212	0.005
Preoperative serum creatinine level (mg/dl)	0.154	0.035, 0.272	0.011
Preoperative serum albumin level (g/dl)	-0.136	-0.243, -0.028	0.014
Postoperative body weight gain (%)	0.037	0.006, 0.068	0.019
Maximal SOFAc score ≥ 3	0.106	0.074, 0.137	< 0.001

CI = confidence interval; RPF = renal-protective fluid management; EuroSCORE = European System for Cardiac Operative Risk Evaluation; SOFAc = cardiovascular sequential organ failure assessment in the first 24 hours.

eTable 7: Multivariable predictors for prolonged length of stay in hospital after surgery.

Predictor	Beta	95% CI	*P* value
RPF group	-0.108	-0.167, -0.048	< 0.001
Hypertension	0.083	0.026, 0.140	0.004
EuroSCORE (logistic)	0.014	0.007, 0.021	< 0.001
Preoperative diuretics use	0.109	0.037, 0.182	0.003
Preoperative serum albumin level (g/dl)	-0.162	-0.233, -0.091	< 0.001
Maximal SOFAc score ≥ 3	0.033	0.013, 0.054	0.002

CI = confidence interval; RPF = renal-protective fluid management; EuroSCORE = European System for Cardiac Operative Risk Evaluation; SOFAc = cardiovascular sequential organ failure assessment in the first 24 hours.

eTable 8: Comparisions of results for primary analysis of acute kidney injury outcome compared with sensitivity analyses.

	RPF strategy	
	Adjusted Odds Ratio (95% CI)	*P* value
Primary model	0.310 (0.191–0.504)	< 0.001
Adjusted for cerebrovascular disease	0.318 (0.195–0.517)	< 0.001
Adjusted for liver disease	0.312 (0.192–0.508)	< 0.001
Adjusted for cerebrovascular disease and liver disease	0.313 (0.192–0.510)	< 0.001
Intraoperative variables only	0.265 (0.164–0.426)	< 0.001
Intraoperative variables only with Maximal SOFAc score	0.278 (0.173–0.449)	< 0.001
Intraoperative variables only with pRBC transfusion	0.308 (0.189–0.499)	< 0.001
subgroup with perioperative cumulative amounts of HES < 30 ml/kg*	0.174 (0.102–0.298)	< 0.001

All models except subgroup model are adjusted age, diabetes mellitus, hypertension, coronary angiography < 5d, preoperative hematocrit, preoperative serum creatinine, preoperative serum albumin, Maximal SOFAc score (except intraoperative variable model), and pRBC transfusion (except intraoperative variable model).

* adjusted diabetes mellitus, hypertension, preoperative serum creatinine, preoperative serum albumin, Maximal SOFAc score, and pRBC transfusion.

RPF = renal-protective fluid management; CI = confidence interval; SOFAc = cardiovascular sequential organ failure assessment in the first 24 hours; pRBC = Packed red blood cell.

eTable 9: Unadjusted and adjusted relationships between perioperative cumulative amounts (ml/kg weight) of HES and postoperative acute kidney injury.

Analysis	Adjusted Odds Ratio (95% CI)	*P* Value
Unadjusted in overall population	1.013 (1.003–1.023)	0.008
Multivariable adjusted in overall population*	1.018 (1.006–1.030)	0.002
Unadjusted in saline group	0.989 (0.976–1.002)	0.106
Multivariable adjusted in saline group†	0.988 (0.973–1.003)	0.106
Unadjusted in RPF group	0.990 (0.943–1.040)	0.696
Multivariable adjusted in RPF group‡	0.987 (0.937–1.041)	0.634

* adjusted diabetes mellitus, hypertension, logistic EuroSCORE, coronary angiography < 5d, preoperative hematocrit, preoperative serum creatinine, preoperative serum albumin, preoperative serum uric acid, diuretics use, anesthesia time, Maximal SOFAc score, and pRBC transfusion.

† adjusted diabetes mellitus, logistic EuroSCORE, coronary angiography < 5d, preoperative hematocrit, preoperative serum albumin, preoperative serum uric acid, and pRBC transfusion.

‡ adjusted age, preoperative serum creatinine, and Maximal SOFAc score.
